# Cerebrospinal fluid cell count variability is a major confounding factor in external ventricular drain-associated infection surveillance diagnostics: a prospective observational study

**DOI:** 10.1186/s13054-021-03715-1

**Published:** 2021-08-11

**Authors:** Marcus Bådholm, Jonas Blixt, Martin Glimåker, Anders Ternhag, Jonas Hedlund, David W. Nelson

**Affiliations:** 1 Function Perioperative Medicine and Intensive Care, Karolinska University Hospital, Stockholm, Sweden; 2 Department of Physiology and Pharmacology, Karolinska Institute, Stockholm, Sweden; 3 Department of Infectious Diseases, Karolinska University Hospital, Stockholm, Sweden; 4 Division of Infectious Diseases, Department of Medicine, Karolinska Institute, Stockholm, Sweden

**Keywords:** External ventricular drain, External ventricular drain associated infections, Infection diagnostics, Cerebrospinal fluid, Cell counts

## Abstract

**Background:**

External ventricular drain (EVD)-related infections (EVDIs) are feared complications that are difficult to rapidly and correctly diagnose, which can lead to unnecessary treatment with broad-spectrum antibiotics. No readily available diagnostic parameters have been identified to reliably predict or identify EVDIs. Moreover, intraventricular hemorrhage is common and affect cerebrospinal fluid (CSF) cellularity. The relationship between leukocytes and erythrocytes is often used to identify suspected infection and triggers the use of antibiotics pending results of cultures, which may take days. Cell count based surveillance diagnostics assumes a homogeneous distribution of cells in the CSF. Given the intraventricular sedimentation of erythrocytes on computed tomography scans this assumption may be erroneous and could affect diagnostics.

**Aims:**

To evaluate the consistency of cell counts in serially sampled CSF from EVDs, with and without patient repositioning, to assess the effect on infection diagnostics.

**Methods:**

We performed a prospective single-center study where routine CSF sampling was followed by a second sample after 10 min, allocated around a standard patient repositioning, or not. Changes in absolute and pairwise cell counts and ratios were analyzed, including mixed regression models.

**Results:**

Data from 51 patients and 162 paired samples were analyzed. We observed substantial changes in CSF cellularity as the result of both resampling and repositioning, with repositioning found to be an independent predictor of bidirectional cellular change. Glucose and lactate levels were affected, however clinically non-significant. No positive CSF cultures were seen during the study. Thirty percent (30%) of patients changed suspected EVDI status, as defined by the cell component of local and national guidelines, when resampling after repositioning.

**Conclusions:**

CSF cell counts are not consistent and are affected by patient movement suggesting a heterogeneity in the intraventricular space. The relationship between leukocytes and erythrocytes was less affected than absolute changes. Importantly, cell changes are found to increase with increased cellularity, often leading to changes in suspected EVDI status. Faster and more precise diagnostics are needed, and methods such as emerging next generation sequencing techniques my provide tools to more timely and accurately guide antibiotic treatment.

*Trial Registration* NCT04736407, Clinicaltrials.gov, retrospectively registered 2nd February 2021.

**Supplementary Information:**

The online version contains supplementary material available at 10.1186/s13054-021-03715-1.

## Background

The external ventricular drain (EVD) is an essential device in the treatment of the neurocritically ill, however, it is associated with a number of complications including EVD related infections (EVDIs) [1]. EVDIs are severe complications, associated with prolonged hospital stay, increased morbidity, and mortality, and may impact long term sequelae [2, 3]. EVDIs are frequently caused by pathogens commonly colonizing the skin at the insertion site [4, 5]. Several studies have shown that the use of hygienic routines in the placement and management of EVDs reduces infection rates, suggesting that bacteria are often introduced introgenically, or by extraluminal migration along the catheter [6–8].

In the literature, the reported incidence of EVDIs ranges between 1 and 35% of inserted drains [9–12]. This disparity results from the lack of an international consensus on a definition for EVDIs and the use of various diagnostic criteria across studies [6]. In a neurointensive care setting, clinical symptoms are often difficult to evaluate due to underlying illness [1, 4, 13]. Consequently, in case of suspicion of EVDI, cerebrospinal fluid (CSF) analysis of glucose, lactate, protein, and cell counts are performed with results within hours [14, 15]. CSF bacterial cultures are the golden standard to verify infection, and positive cultures or bacterial identification using 16S sequencing often means true infection but could also be a result of contamination, thus, true positives can be difficult to distinguish based on the cultured bacteria alone [16, 17]. In the neurocritically ill, CSF parameters are frequently influenced by intraventricular hemorrhage (IVH) and CSF cell counts in particular may vary greatly depending on the IVH volume [18]. CSF bacterial cultures may take several days to finalize and prior use of antibiotics may further prolong culture incubation time or cause false negative cultures [13]. Thus, it is seldom possible to delay treatment until the bacterial cultures have finalized [8]. Ideally the clinical picture would help in rejecting false positives, however, parameters and symptoms are often ambiguous.

The cell index has been suggested as a method to adjust for confounding caused by IVH in EVDI surveillance diagnostics, where the ratio of leukocytes to erythrocytes in CSF (LE ratio) is compared to the ratio in peripheral blood [18, 19]. However, the LE ratio fails to account for the fact that the blood causes an aseptic inflammation, resulting in immigration of leukocytes [1, 18, 20]. Furthermore, a greater aseptic response is elicited in patients with greater intraventricular blood volumes [21]. Cell count based metrics, the LE ratio included, are based on the assumption that erythrocytes and leukocytes are homogeneously distributed in the CSF. However, computed tomography (CT) scans frequently show intraventricular gravity sedimentation of blood, suggesting that a homogeneous distribution is unlikely. Leukocytes and erythrocytes possesses different densities which causes them to separate during gravity sedimentation [22, 23]. With the densities of CSF and blood plasma being similar, intraventricular gravity sedimentation should in theory mimic that of gravity sedimentation of whole blood, with leukocytes positioned on top of the erythrocytes in analogy to a buffy coat. Additionally, leukocytes and in particular granulocytes, exhibit anti-sedimentation, a property that have been shown to be exacerbated in critically ill patients, thus, leukocytes may sediment at a slower rate in this patient group [24–26]. As such, leukocytes may be more susceptible to turbulence or intraventricular shifting compared to erythrocytes.

In summary, due to the pathogens involved, patient characteristics, and confounding factors, early identification of EVDIs have proven difficult and no CSF parameter, by itself or in aggregate, have shown to reliably predict or identify EVDIs [5, 27, 28]. Combined with the potential severity of EVDIs, this has led to an excessive use of broad-spectrum antibiotics in this patient group [8, 13]. Methods to adjust for IVH are based on the uncertain assumption that cells are homogeneously distributed in the CSF. If, and how, this affects routinely analyzed CSF parameters has yet to be studied.

We hypothesize that a heterogeneous distribution of blood components in the CSF may affect the variability of routinely analyzed CSF parameters. The aim of this study was to test this hypothesis by testing the reproducibility of serially drawn CSF samples and to evaluate if resampling and changes in a patient’s body position affects CSF parameters.

## Methods

### Setting and participants

**Fig. 1:**
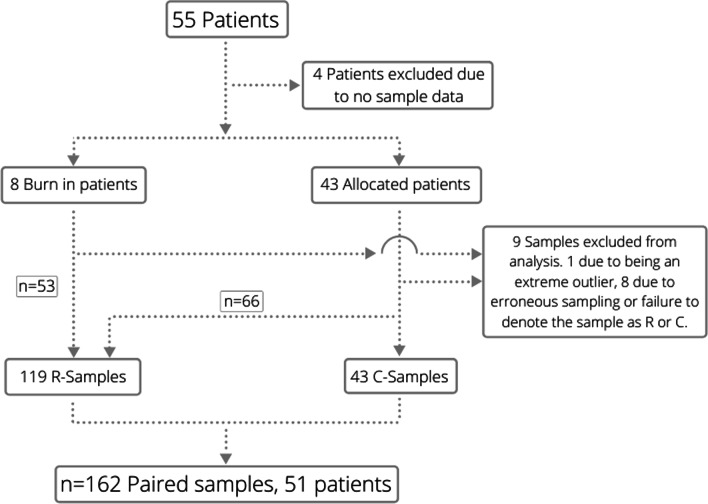
Study flowchart. All burn-in patients were repositioned (R) before resampling. All others were allocated to R or Control (C) with a 10 min wait time before resampling but no repositioning

This prospective observational single-center study with allocation, included adult (18 or older) patients treated with EVDs at the neurointensive care unit (NICU) at the Karolinska University Hospital in Stockholm. Exclusion criteria were patients admitted with bacterial or viral CNS infections. The data collection process is shown in Fig. 1.

### Data collection and intervention

Two CSF samples (paired samples) were collected biweekly or during workup due to suspected infection as per routine. The first eight patients were considered burn-in patients in order to familiarize the staff with the study and to identify any problems related to the sampling routine. All paired samples were allocated individually to two serially drawn samples performed before and after a standard clinical repositioning (repositioned samples, R-samples), or a 10-min wait period between two serially drawn samples (control samples, C-samples). As such, individual patients could have both R-samples and C-samples. For R-samples, patients were positioned in a lateral side position two hours prior to sample 1. After the first sample, patients were repositioned to the contralateral side after which the second sample was collected. For C-samples, patients were stationary 2 h prior to sample 1 and 10 min separated the two samples, corresponding to the average time between R-samples, as identified during the burn-in period. Non-sedated and compliant patients were asked to lie in a lateral side position prior to the first sample in applicable cases. Open drains were closed 5 min prior to the first sample and remained so between samples. 1.5 mL of CSF was drawn and discarded prior to each sample. After the burn-in period of eight patients with only R-samples, all patients were allocated to R-samples or C-samples for each sample session. CSF was analyzed for cell counts, lactate, glucose, and albumin by the Karolinska University Hospital Laboratory. The laboratory utilizes machine cell counting using the Sysmex XN10 system. Sampling was performed by a registered nurse. Information on primary diagnosis, length of stay, sex, Glascow Coma Scale (GCS), GCS motor response, and antibiotic treatment were collected. The intraventricular blood volume was measured from CT scans using the MPR volume tool in the Sectra IDS7 system (version 21.1.1.1997) as well as estimated using the intraventricular hemorrhage score (IVHS) by Hallevi et al. [29]. The shortest two-dimensional distance between the tip of the EVD catheter and intraventricular blood was measured, as well as the depth of blood sediment in the dorsal horns of the lateral ventricles. The measured IVH volume, IVHS, the two-dimensional catheter distance, and the sediment depth were measured or estimated using the earliest CT scans after EVD insertion. For IVHS calculation see Additional file 2.

### EVDI definition

The cell count components of the Karolinska University Hospital (earlier Swedish national guideline) EVDI definitions were used to denote suspected EVDI status for each individual sample as suspected EVDI (SI) or no EVDI (NI) to identify clinical implications of any observed CSF cell count variability. The cell count component of our local guidelines is one of several metrics used to identify suspected infections and guide antibiotic treatment in wait of culture results. The cell count component of the new current Swedish national guidelines was also tested for the same purpose and is included below [30].

#### Cell count component of Karolinska University Hospital and earlier national EVDI definition

1. CSF cellular constitution consistent with EVDI as follows:
  $$\begin{aligned} Granulocytes-\frac{Erythrocytes}{1000}>100 \end{aligned}$$

#### Cell count component of the current national EVDI guidelines

1. CSF-Leukocytes $>250*10^6$/L with predominantly granulocytes.

### Derived variables

Derived variables are defined as below. $\Delta$ is used to denote the difference between paired samples (sample 1 and sample 2) for all CSF parameters. The absolute value (nonnegative) is denoted by two vertical lines (|*x*|).

$$\begin{aligned} \Delta \;=\;Cell\;count\;2 - Cell\;count\;1 \end{aligned}$$

A positive $\Delta$ indicates an increase from sample 1 to sample 2.

$$\begin{aligned} Relative\;\Delta \;=\;\frac{Cell\;count\;2 - Cell\;count\;1}{Cell\;count\;1} \end{aligned}$$

The relative cell count $\Delta$ is presented as percentage (IQR).

$$\begin{aligned} Absolute\;\Delta \;=\;|Cell\;count\;2 - Cell\;count\;1| \end{aligned}$$

A positive and negative $\Delta$ will here have the same number.

$$\begin{aligned} LE\;Ratio\;=\;\frac{Granulocytes}{Erythrocytes}*1000 \end{aligned}$$

### Statistical analysis

Continuous variables were described as median, interquartile range (IQR), and percentage. The LE ratio was calculated for samples with cell count levels above the lower limit of the Sysmex XN10 system (Additional file 1), as measurement imprecision could produce false positive changes in the LE ratio at lower cell counts. The nonparametric *Clustered Wilcoxon Signed Rank test* was used to compare the median location shift for each CSF parameter between sample 1 and 2 [31, 32], clustered by Patient ID. The nonparametric *Fligner-Killeen test* was used to compare the variance of the derived variables between R-samples and C-samples. *Mixed effects linear regression analysis* was used to identify correlations between CT-derived variables and the pair-wise differences in a univariate setting with Patient ID as a random effect with random intercept, but not slope. *Mixed effects logistic regression analysis* was used to evaluate the effect of repositioning on pair-wise differences and to identify variables that were correlated to changes in suspected EVDI status between samples, also with Patient ID as random intercept but not slope. Outliers were identified based on Cook’s distance and removed. Fifteen R-samples and 5 C-samples had CSF cell counts below the functional sensitivity of the Sysmex XN10 system and were excluded from the mixed effects regression analyses and LE ratio calculations. Thus, 104 R-samples and 38 C-samples had the LE ratio calculated and were included in the mixed effects regression analyses.

All data were analyzed and allocation was performed using computer generated randomization in the statistical program R version 4.0.3. Mixed effects regression analyses were performed using R package lme4 v1.1-21. A *p*-value of $p<.05$ is considered significant in regression analyses. In tables with multiple comparisons, *p*-values of $p<.01$ are bolded.

## Results

### Demographics

**Fig. 2:**
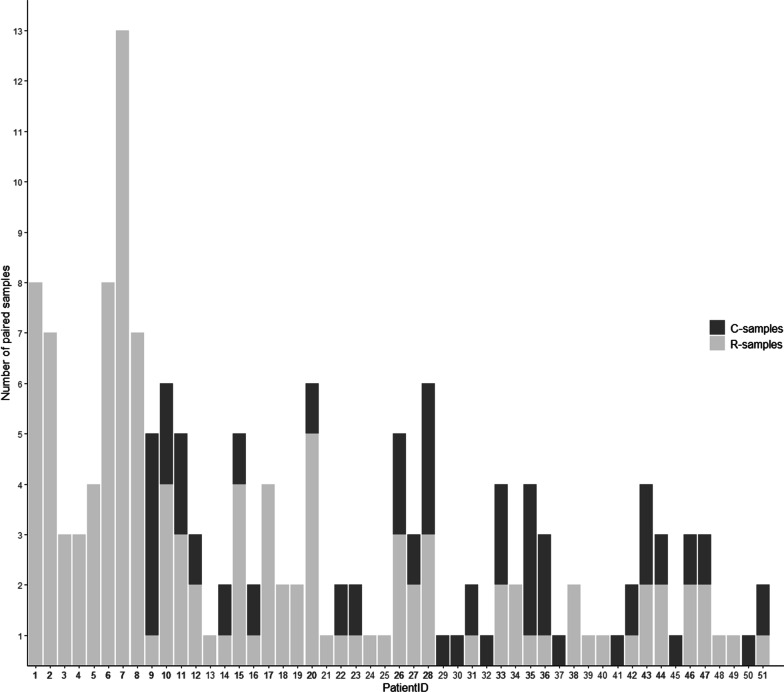
Paired samples per patient. R-samples (light gray) and C-Samples (dark gray) per patient. The first eight patients were all repositioned before resampling (burn-in period). The number of samples per patient was influenced by length of stay which varied significantly between patients

The mean age of the cohort was 61 years and 61% were female (Table 1). The dominant NICU admission diagnosis was subarachnoidal hemorrhage (80%). Sample groups and numbers are depicted (Fig. 1). One hundred sixty-two paired samples were included in the final analysis, 119 R-samples and 43 C-samples. The difference between the two groups was in part attributable to the burn-in period of eight patients and differences in length of stay between patients, contributing to large interindividual differences in the number of samples per patient (Fig. 2).

**Table 1:** Patient characteristics

Characteristics	
Patients	51
Male sex	20 (39)
Age	61 (49, 70)
Length of stay, days	14 (10, 18.5)
Paired samples	162
R-samples	119 (73.5)
C-samples	43 (26.5)
Patients allocated to R-samples	44 (86)
Changed EVDI status	13 (29.5)
Patients allocated to C-samples	30 (59)
Changed EVDI status	0 (0)
CT-derived variables	
IVH volume, mm^3^	1210 (223, 6526)
Sediment depth, mm	16 (9.75, 27)
Catheter distance, mm	17.5 (2.5, 48)
IVHS, score 1–23	10 (8, 11)
Admission diagnoses	
SAH	41
ICH	7
Trauma	3

Data are presented in no (%), or median (IQR). Percentage allocated to R-samples or C-samples represent percentage of whole patient cohort. IVH volume, Sediment Depth, Catheter Distance, and IVHS are defined as in methods. EVDI: external ventricular drain infection. R-samples: paired samples around a patient repositioning. C-samples: paired non-repositioned samples. IVH: intraventricular hemorrhage. IVHS: intraventricular hemorrhage score. SAH: subarachnoid hemorrhage. ICH: intracerebral hemorrhage

**Table 2:** Comparison of CSF variable variances between repositioned and non-repositioned samples and medians between serial samples

	R-samples	C-samples	*P*-value	Statistical test
*2.1 Outcome Variance R-samples versus C-samples*				
Granulocytes, $10^6$				
$\Delta$, Range	− 3505 , 619	− 77 , 757		
$\Delta$, IQR	[− 4.5 , 50.5]	[− 1 , 2.5]	$<{\mathbf .001}$	Fligner Killeen
Relative $\Delta$, IQR	[− 31 , 80]	[− 10 , 15]	$<{\mathbf .001}$	Fligner Killeen
Absolute $\Delta$, IQR	[3 , 95.5]	[0 , 8.5]	$<{\mathbf .001}$	Fligner Killeen
Erythrocytes, $10^6$				
$\Delta$, Range	− 271,500 , 331,700	− 47,500 , 121,100		
$\Delta$, IQR	[− 2450 , 13,150]	[− 400 , 2450]	$<{\mathbf .001}$	Fligner Killeen
Relative $\Delta$, IQR	[− 23 , 56]	[− 10 , 20]	$<{\mathbf .001}$	Fligner Killeen
Absolute $\Delta$, IQR	[900 , 20,050]	[250 , 4950]	$<{\mathbf .001}$	Fligner Killeen
Albumin, mg/L				
$\Delta$, Range	− 222 , 603	− 68 , 378		
$\Delta$, IQR	[− 3 , 72]	[− 1 , 28]	$<{\mathbf .01}$	Fligner Killeen
Relative $\Delta$, IQR	[− 1.9 , 20]	[− 0.5 , 11]	.02	Fligner Killeen
Absolute $\Delta$, IQR	[8 , 90.5]	[3 , 30.5]	$<{\mathbf .001}$	Fligner Killeen
Lactate, mmol/L				
$\Delta$, Range	− 1.1 , 0.8	− 0.3 , 0.4		
$\Delta$, IQR	[− 0.1 , 0.1]	[0 , 0.05]	$<{\mathbf .01}$	Fligner Killeen
Relative $\Delta$, IQR	[− 3.2 , 4]	[0 , 1.1]	$<{\mathbf .01}$	Fligner Killeen
Absolute $\Delta$, IQR	[0.1 , 0.2]	[0 , 0.1]	.1	Fligner Killeen
Glucose, mmol/L				
$\Delta$, Range	− 0.6 , 0.8	− 1.6 , 0.2		
$\Delta$, IQR	[− 0.2 , 0.1]	[− 0.1 , 0]	$<{\mathbf .01}$	Fligner Killeen
Relative $\Delta$, IQR	[− 2.7 , 2.4]	[− 2.1 , 0]	$<{\mathbf .01}$	Fligner Killeen
Absolute $\Delta$, IQR	[0.1 , 0.2]	[0 , 0.1]	.04	Fligner Killeen
LE Ratio				
$\Delta$, Range	− 72.1 , 40.1	− 2.1 , 3.9		
$\Delta$, IQR	[− 0.55 , 0.64]	[− 0.25 , 0.38]	.02	Fligner Killeen
Relative $\Delta$, IQR	[− 15 , 35]	[− 19 , 17]	.06	Fligner Killeen
Absolute $\Delta$, IQR	[0.15 , 2]	[0.066 , 1.3]	$<{\mathbf .01}$	Fligner Killeen

	R-Samples			C-Samples			
	Sample 1	Sample 2	*P*-value	Sample 1	Sample 2	*P*-value	Statistical test
*2.2 Outcome Median Sample 1 versus Sample 2*							
Granulocytes, $10^6$	40	54	.33	13	14	.81	Clustered Signed rank
Erythrocytes, $10^6$	22,100	22,300	.25	7700	6700	.37	Clustered Signed rank
Albumin, mg/L	318	333	$<{\mathbf .001}$	235	251	.02	Clustered Signed rank
Lactate, mmol/L	3.2	3.3	.56	3.1	3.1	.53	Clustered Signed rank
Glucose, mmol/L	4.5	4.5	.19	4.5	4.5	.35	Clustered Signed rank
LE ratio	2.16	2.24	.25	2.51	2.31	.76	Clustered Signed rank

Data are presented as IQR (square brackets) and in the case of the $\Delta$, the range is also included (no brackets) in 2.1. Data are presented as medians in 2.2. Sample medians were evaluated with the clustered Wilcoxon signed rank, clustered by Patient ID. Variances were evaluated with the Fligner–Killeen test. The results of $p<.01$ are bolded. As patients are represented in the R-samples and C-samples unequally, mixed effects regression models are required for further interpretation of this data. $\Delta$: pair-wise difference, including absolute and relative derivatives. R-samples: Repositioned samples. C-Samples: Non-repositioned control samples

### CSF variables

Substantial pair-wise differences were seen in both R- and C-samples for granulocytes (absolute $\Delta$ IQR [3, 95.5] and [0, 8.5], respectively) and erythrocytes (absolute $\Delta$ IQR [900, 20,050] and [250, 4950], respectively), where the variability of drawn samples suggests a potential to confound early identification of EVDIs (Table 2.1). The IQR does not clearly reflect the large variability seen in this study as many patients had low cell counts. While we expected cells to be sedimented and affected in a positive direction, changes were both negative and positive in equal amounts (bidirectional), resulting in no significant differences in mean or medians between paired samples on a cohort level ($p > 0.05$, clustered signed rank) (Table 2.2), with the exception of albumin. This suggests a heterogeneous sample space.

**Fig. 3:**
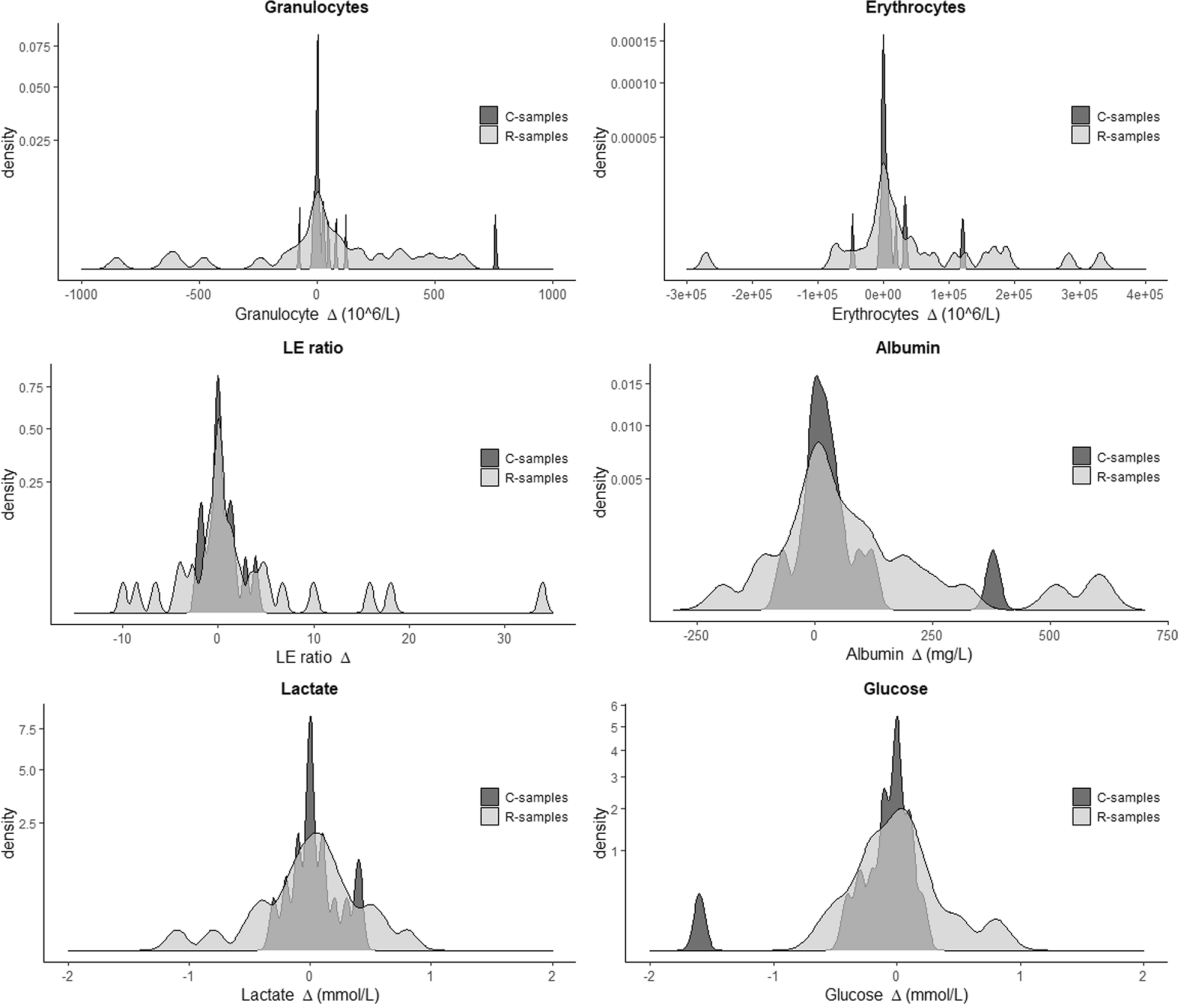
Variability of paired samples. Density plots of the pair-wise $\Delta$ distributions for R-samples (light gray) and C-samples (dark gray) for all CSF parameters, respectively. The x-axis shows the pair-wise differences or $\Delta$. The area under each will be sum-able to 1, defining the y-axis values. One R-sample with a $\Delta$ of − 3505 was excluded from the granulocyte plot in order to improve visualization. R-samples: paired samples around a patient repositioning. C-samples: paired non-repositioned samples

The variance of granulocytes (range R-samples [− 3505, 619] and C-samples [− 77, 757]) and erythrocytes (range R-samples [− 271,500, 331,700] and C-samples [− 47,500, 121,100]) was significantly greater ($p<.01$, Fligner Killeen) (Table 2.1, Fig. 3) in the R-samples, suggesting that movement affect variability, again bidirectional. The correlation between repositioning and cell count $\Delta$’s and derivatives were evaluated with logistic regression mixed model analyses with Patient ID as a random effect (Table 3). The absolute $\Delta$, but not the $\Delta$ or relative $\Delta$, were significantly related to repositioning for all variables, supporting that the variance is greater in the repositioned group, but that the direction of change is random ($p<.05$, Table 3). To summarize, the exacerbated CSF cell count variability seen in the R-samples was significantly correlated to repositioning of the patient in a mixed effects setting (Table 3), and not merely an effect of sample group heterogeneity.

**Table 3:** Effect of repositioning

Predictor variables	Repositioned
	*P*-value
LE ratio	
$\Delta$*	.1
Relative $\Delta$	.04
Absolute $\Delta$*	$<{\mathbf .01}$
Granulocytes	
$\Delta$*	.09
Relative $\Delta$	.046
Absolute $\Delta$*	$<{\mathbf .001}$
Erythrocytes	
$\Delta$*	.61
Relative $\Delta$	.1
Absolute $\Delta$*	$<{\mathbf .001}$
Albumin	
$\Delta$*	.06
Relative $\Delta$	.12
Absolute $\Delta$*	.01
Lactate	
$\Delta$	.64
Relative $\Delta$	.36
Absolute $\Delta$	$<{\mathbf .01}$
Glucose	
$\Delta$	.78
Relative $\Delta$	.93
Absolute $\Delta$	$<{\mathbf .01}$

Univariate mixed effect logistic regression analysis toward repositioning. The results of $p<.01$ are bolded. Changes in all parameters can be seen attributable to repositioning, but not unidirectional and therefore predominately in the absolute $\Delta$ representation. *indicates log transformation of variable. $\Delta$: pair-wise difference, including absolute and relative derivatives

**Fig. 4:**
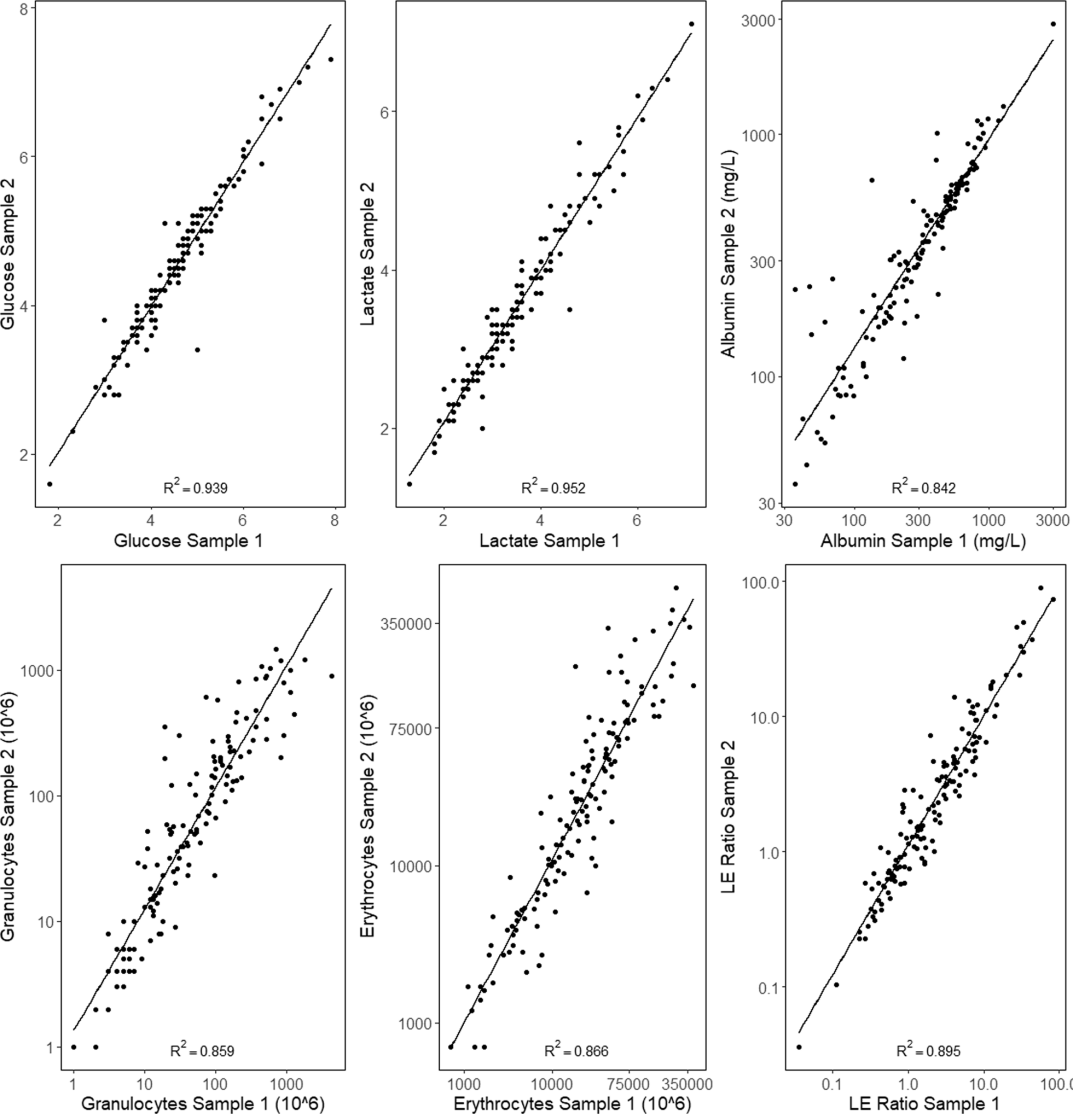
Correlations of paired samples. Scatter plots comparing sample 1 on the x-axis with sample 2 on the y-axis for each CSF parameter. The $R^2$ of linear regression is given. The correlations are seen weaker for cells exhibiting a greater variability of paired samples

**Fig. 5:**
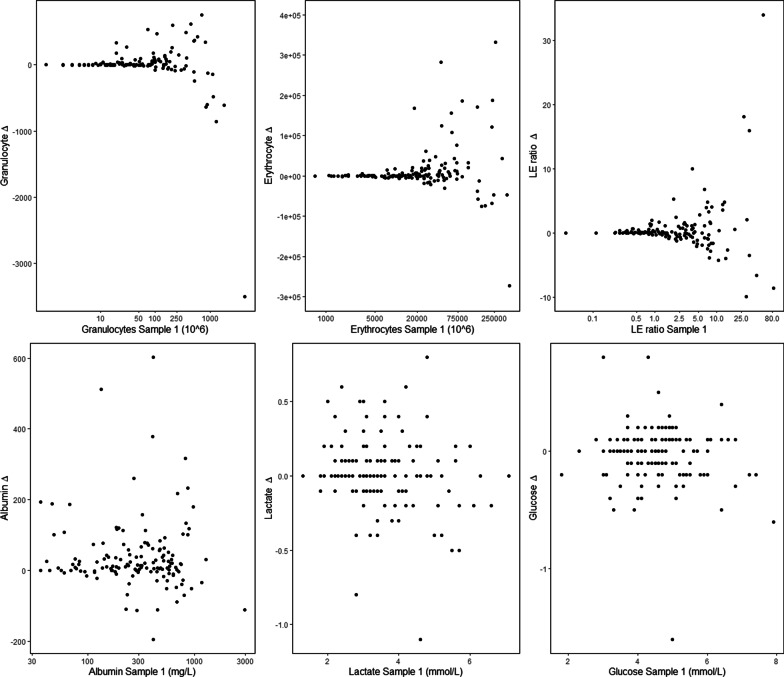
Variability in relation to initial levels. Plots of sample 1 levels versus the pair-wise $\Delta$ for each CSF parameter. Albumin, granulocytes, erythrocytes, and the LE ratio have been log-transformed in order to improve visualization. Cells show a clear heteroscedasticity potentially impacting diagnostics that is not seen for glucose and lactate, but to some extent for albumin. This suggests a heterogeneity of larger molecules in the sampled volumes. $\Delta$: pair-wise difference

Cell counts varied more compared to glucose, lactate, and albumin, which exhibited an intermediate level, suggesting that molecular weight or size may also influence variability (Fig. 4). Here, the paired LE ratio is more highly correlated than erythrocytes and leukocytes individually, suggesting that changes may be in concert between cell types. However, the correlation between paired samples of monocytes and granulocytes is stronger than that of leukocytes and erythrocytes (Additional file 3), suggesting that cell types are differentially distributed in the sample space. Pair-wise variability increased with increasing levels in sample 1 for cells in particular (Fig. 5), but not glucose and lactate, consequently cells showed larger variability at levels used for clinical EVDI cutoffs for suspected infection. In conclusion, CSF cell counts varied significantly and could not be reproduced in serially drawn CSF, where R-samples exhibited greater variability.

### Cell count criteria for suspected EVDI

When the Stockholm EVDI cell count criteria for suspected infection were applied on our cohort as to evaluate changes of suspected EVDI status (suspected infection, SI, or no infection, NI) between paired samples, 14 R-samples from 13 unique patients changed EVDI diagnostic group, corresponding to 30% of all patients with collected R-samples in our cohort. Eleven R-samples changed from NI to SI, and 3 R-samples from SI to NI. One patient had changes in both directions with one paired sample that changed from NI to SI, and one that changed from SI to NI. No changes in suspected EVDI status were seen for any C-samples. Two patients were initiated on broad-spectrum antibiotics due to significant pleocytosis in the second sample. No patient had a positive CSF culture confirmed EVDI in this study.

Repositioning as a cause of suspected EVDI group change could not computationally be evaluated with logistic regression as all positive events occurred solely for R-samples. Repositioning was, however, significantly correlated with suspected EVDI group change in a chi-squared test ($p=.042$) suggesting that cell changes in regions near diagnostic cutoffs may be influenced by patient movement. The cell count criteria from the new national EVDI guidelines applied to the sample cohort resulted in 18 R-samples and 2 C-samples from 16 unique patients changing diagnostic status ($p=.06$) in logistic regression. No random effects were seen for patient ID. No other studied parameter was found significantly correlated with changes of suspected EVDI status (Table 4). In summary, 30% of all patients with R-samples changed diagnostic EVDI status at least once during the study period, indicating that EVDI surveillance diagnostics based on cell counts are neither sensitive nor specific in a clinical setting.

**Table 4:** Changes of diagnostic status and suspected infection

Predictor variables	Diagnostic EVDI status changed	
	*P*-value	
Repositioned	NA	
Erythrocytes 1	.4	
Leukocytes 1	.74	
Monocytes 1	.82	
Granulocytes 1	.73	
Glucose 1	.61	
Lactate 1	.6	
Albumin 1	.74	
LE ratio 1	.47	
Sample number	.58	
Catheter distance	.51	
Sediment depth	.9	
IVHS	.55	
IVH volume	.24	
GCS	.25	
GCS motor	.16	

Univariate logistic mixed model analyses toward diagnostic EVDI status change. No variable can be seen to predict change of status. The variable repositioning could, however, not be included in this analysis computationally due to zero events in the C-samples, thus noted not applicable (NA), but was seen statistically significant ($p=.042$) with a Chi-Squared test. IVH volume, sediment depth, catheter distance, and IVHS are defined in the methods section. IVHS: Intraventricular Hemorrhage Score, see methods section. EVDI: External Ventricular Drain Infection. GCS: Glascow Coma Scale. GCS motor: Glascow Coma Scale motor response

### CT-derived variables

Catheter distance, sediment depth, measured blood volume, and IVHS were calculated or estimated based on the first CT scan after EVD insertion. The correlation between all CT-derived variables and the observed $\Delta$’s and derivatives for all CSF parameters were evaluated in a univariate mixed effects regression model with Patient ID as a random effect. IVH volume was not seen significantly related to any CSF variable. Other CT parameters appear most highly related to erythrocyte-derived variables (Additional file 4), but not the direction of change. However, no significance here withstands adjustment for multiple testing and the explanatory value is found limited with no pseudo $R^2$ above 0.0635 for the fixed effects.

## Discussion

In this study we tested the reproducibility of two serially drawn CSF samples in patients who were allocated to sampling around a routine patient repositioning, or not. We show that serially drawn CSF parameters, and cell counts in particular, were subject to significant variability which may greatly influence early identification of EVDIs and impact clinical decision making . Additionally, patient movement was also shown to effect cell counts and diagnostics.

Homogeneity of the sample space is assumed in EVDI surveillance diagnostics but has not been tested previously. Gravity sedimentation of erythrocytes is seemingly apparent from CT imaging in these patients but the effect on sampled cell counts is unclear. The effect of cellular sedimentation in the CSF has been studied previously, but only relating to lumbar versus ventricular drains.

Previous research and case studies have found cellularity to be increased in lumbar CSF compared to ventricular CSF [33–36]. Additionally, both Podkovik et al and Gerber et al noted the risk of these findings impacting early infection diagnostics [34, 35]. As a sedimentation effect that may impact clinical decision making have been demonstrated for the lumbar versus the ventricular region, it is somewhat surprising that the local sedimentation effect in the ventricles have not been previously studied.

This study provides insight to the assumption of homogeneity. We found substantial cell variability between serially drawn CSF samples with the largest difference observed being -3505*10^6^ granulocytes in the second sample, suggesting an initial heterogeneity in the sample space. However, in opposition with our initial hypothesis that there would be a predominate increase of leukocytes (due to potential turbulence caused by initial sampling or the effect of repositioning), changes were generally bidirectional. Bidirectional changes make theoretical sense, as blood can be shifted both away and toward the catheter in the intraventricular space. Repositioning, and thus patient movement, was also found significantly related to changes in cell count, further compounding that of serial sampling. Moreover, changes, and direction of changes, of cell levels could not be readily predicted from our CT-derived variables, further suggesting that an initial heterogeneity of the sample space leads to random direction of change. In aggregate, it is clear from this study that a basic assumption of EVDI surveillance diagnostics that cells are homogeneously distributed in the CSF is erroneous and that resampling and a simple routine patient repositioning both result in changes in sampled CSF cell counts, impacting early identification of EDVIs.

The potential impact of variable CSF cell counts on EVDI surveillance diagnostics is readily seen in this study where 30% of repositioned patients changed EVDI diagnostic status in the course of 10 min based on the cell count criteria for suspected infection. We also noted that variability increased with increasing cell counts levels (heteroscedasticity) such that large changes are found near to diagnostic cutoff levels. This heteroscedasticity, as shown in Fig. 5, is apparent for cells but not for lactate and glucose, with albumin showing an intermediate nature, suggesting that molecular size or weight may be related to homogeneity. Generally, serial changes in lactate and glucose are found small and clinically insignificant and may represent more stable components of diagnostic algorithms. In summary, variability of cell counts, especially near cutoffs used for EVDI surveillance diagnostics are highly impacted by sample space heterogeneity.

The LE ratio, or variations thereof based on the rationale that the number of leukocytes is related to the amount of blood in the non-infected state, is one of the methods used in EVDI surveillance diagnostics. We observed significant changes of the LE ratio when resampling. The changes in cell counts were bidirectional and independent between cell types. Repositioning exacerbated changes in the LE ratio, but we were not able to predict neither the magnitude nor the direction of the change which makes any variability difficult to adjust for in a clinical setting. While changes in the LE ratio were more stable compared to individual cell types, the variability increased here also with higher ratios in the first sample (Fig.  5). Appropriate LE ratio cutoffs have been difficult to establish with satisfactory sensitivity and specificity for early EVDI identification. The cell index have been suggested to improve sensitivity and specificity for EVDI surveillance diagnostics [19]. A CI of 10 as a cutoff to identify EVDIs early with a sensitivity of 80% and specificity of 75% was suggested by Liew et al. [37]. As they included 95 patients with and without IVH the diagnostic utility of the CI in EVDI surveillance diagnostics in patients with large intraventricular blood volumes remain uncertain. In contrast, Lunardi et al. suggested a vastly different CI of 2.9 as a suspected EVDI cutoff with a sensitivity of 95% and a specificity of 92.9%, in their prospective study of 34 patients [18]. Thus, variability, as seen in our study, elucidates why the utility of the LE ratio, and by extension the cell index, for early EVDI surveillance diagnostics remains uncertain, and why definitive cutoffs have been difficult to establish.

Historically, suspected EVDI and verified EVDI have been difficult to separate prior to CSF bacterial culture results based on common diagnostic EVDI surveillance criteria, in which cell counts are central, resulting in frequent over-treatment with antibiotics [5, 27, 28]. We believe that the CSF cell count variability demonstrated by us in this study may explain the lack of sensitivity and specificity yielded by cell count-based diagnostic surveillance algorithms. Unfortunately, except for a standard patient repositioning and a greater intraventricular blood volume, we were not able to identify a single laboratory, clinical, or demographic variable that could assist in a clinical setting to predict neither the magnitude nor the direction of CSF cellular change. We were also unable to identify predictors of changes in suspected EVDI status, except that those with cell counts close to cutoffs have the greatest risk of change. Neither can we recommend that a patient should not be repositioned two hours before CSF sampling, nor the opposite, that a patient positioning should be performed prior to CSF sampling, as we cannot determine which regime would yield CSF samples that best represent true intraventricular conditions. In summary, this study introduces further complexity into the use of surveillance algorithms using cell counts. We suggest that future studies aim to investigate less cell count centric algorithms or focus on novel techniques for rapid direct bacterial detection.

### Limitations

There are several limitations to this study. This is a single-center clinical trial and the external validity of this study may need further validation. As different centers will have different treatment regimes, routines, and algorithms concerning EVD care and EVDI surveillance diagnostics, findings may be to some extent be population and location dependent. Moreover, we have not studied the time component of cell change, where the aseptic inflammation component might be expected to increase over time, and heterogeneity might itself be related to the inflammatory process. However, as the risk of true infection also increases over time, late variability would also be expected to impact diagnostics. Additionally, our study cohort is 80% SAH patients, and power will not permit if there is a disease-related component to variability. As, we have no positive cultures we provide no information on if heterogeneity is different in true infection. Finally, our sample groups are unbalanced, mainly due to a burn-in period where all patient samples were allocated around repositioning, with by chance long ICU stays. We have attempted to account for this using mixed model analyses.

## Conclusions

Current methods of adjusting for IVH in EVDI surveillance diagnostics are based on the assumption that blood is homogeneously distributed in the CSF and that CSF sampling provides a fair representation of intraventricular blood volumes and granulocyte counts. Our findings in this prospective observational study with allocation to repositioning or not suggest that this assumption is erroneous. CSF cell counts varied greatly in serially drawn samples with repositioning also significantly contributing to change in mixed effect models. This change was seen to be in both directions and is larger at higher counts, confounding EVDI surveillance diagnostics. Importantly, one-third of the patients in our repositioned group changed suspected EVDI status in the time frame of 10 min between CSF samplings based on the cell count criteria from local and national EVDI diagnostic guidelines. Two patients who would otherwise not have been initiated on broad-spectrum antibiotics received treatment due to significant pleocytosis in the second sample. Consequently, analysis of current CSF parameters may not be adequate to distinguish between natural aseptic inflammation and EVDIs in neurocritically ill patients. Our findings add yet more complexity into identifying and defining EVDIs prior to bacterial cultures and may explain why no routinely analyzed CSF parameter, singularly or in aggregate, have been found to reliably predict or identify EVDIs.

Faster and more precise diagnostics are needed, and methods such as emerging next-generation sequencing techniques may provide tools to more timely and accurately exclude EVDIs and guide antibiotic treatment.

## Supplementary Information

**Additional file 1**: Sysmex XN10 machine cell counting.docx: Information regarding Sysmex XN10 machine cell counting and the term functional sensitivity.

**Additional file 2**: Intraventricular Hemorrhage Score (IVHS).docx: Information regarding IVHS calculation.

**Additional file 3**: Correlation of paired sample differences and cell types.PNG: Scatter plots comparing the granulocyte Δ with the erythrocyte Δ and the granulocyte Δ with the monocyte Δ, respectively. Granulocytes and monocytes exhibit a stronger correlation (*R*^2^ = 0,624) vs. granulocytes and erythrocytes (*R*^2^ = 0,346) suggesting that a greater concordance is seen for cells of similar types. Δ: pair-wise difference.

**Additional file 4**: Computed tomography (CT)-related predictors of change between pair samples.PNG: Table with univariate mixed model linear regression analyses. The results of *p* < .01 are bolded. CT parameters appear most correlated with changes in erythrocytes, but not with direction of change. IVH volume, sediment depth, catheter distance, and IVHS are defined in the methods section. Δ: pair-wise difference, including absolute and relative derivatives. IVHS: intraventricular hemorrhage score. IVH: intraventricular hemorrhage.

## Data Availability

The data set used and analyzed are available from the corresponding author on reasonable request.
