# Supplementary material for: Cerebrospinal fluid cell count variability is a major confounding factor in external ventricular drain-associated infection surveillance diagnostics: a prospective observational study
Source: Crit Care. 2021 Aug 11;25:291. doi: 10.1186/s13054-021-03715-1 (PMC8359042; doi:10.1186/s13054-021-03715-1)
Supplement: Supplementary file 1 — Additional file 1: Sysmex XN10 machine cell counting.docx: Information regarding Sysmex XN10 machine cell counting and the term functional sensitivity. [file 13054_2021_3715_MOESM1_ESM.docx]

## Sysmex XN10 Machine Counting

The Karolinska University Hospital laboratory utilizes machine counting with the Sysmex XN10 system. The coefficient of variation (CV) denotes a methods inherent imprecision on serial analyses on identical samples and is defined as:

$$CV(\%)=\frac{Standard Deviation}{Mean *100}$$

The functional sensitivity of a method is defined as the lowest measurable sample concentration with an acceptable CV, this limit is commonly drawn at *CV* = 20% for machine counting of cells in CSF. The functional sensitivity for the Sysmex XN10 is at CSF-leukocytes = 5 ∗ 10^6^/L and CSF-erythrocytes = 300 ∗ 10^6^/L, counts below these limits are susceptible to what is judged as unacceptable measurement imprecision. This information was obtained via direct contact with the Karolinska University Hospital laboratory and their local tests of the Sysmex XN10.
