# Supplementary material for: Cerebrospinal fluid cell count variability is a major confounding factor in external ventricular drain-associated infection surveillance diagnostics: a prospective observational study
Source: Crit Care. 2021 Aug 11;25:291. doi: 10.1186/s13054-021-03715-1 (PMC8359042; doi:10.1186/s13054-021-03715-1)
Supplement: Supplementary file 2 — Additional file 2: Intraventricular Hemorrhage Score (IVHS).docx: Information regarding IVHS calculation. [file 13054_2021_3715_MOESM2_ESM.docx]

## The Intraventricular Hemorrhage Score

The IVHS ranges from 0-23 points based on the amount of blood in the four ventricles and the presence or absence of hydrocephalus. Lateral ventricles were scored 0-3 for no blood, up to one third, a third to two thirds, or two thirds to completely filled with blood. The third and fourth ventricles were scored 0 or 1 for no blood or any amount of blood. Hydrocephalus was scored 0 or 1 for the presence or absence of hydrocephalus [28]. The IVHS was calculated as following:

*IVHS* = 3 ∗ (*RV* + *LV* ) + *III* + *IV* + 3 ∗ *H*

RV = Right lateral ventricle (0-3). LV = Left lateral ventricle (0-3). III = Third ventricle (0, 1). IV = Forth ventricle (0, 1). H = Hydrocephalus (0, 1)
