# Supplementary figures and images for: Cerebrospinal fluid cell count variability is a major confounding factor in external ventricular drain-associated infection surveillance diagnostics: a prospective observational study
Source: Crit Care. 2021 Aug 11;25:291. doi: 10.1186/s13054-021-03715-1 (PMC8359042; doi:10.1186/s13054-021-03715-1)

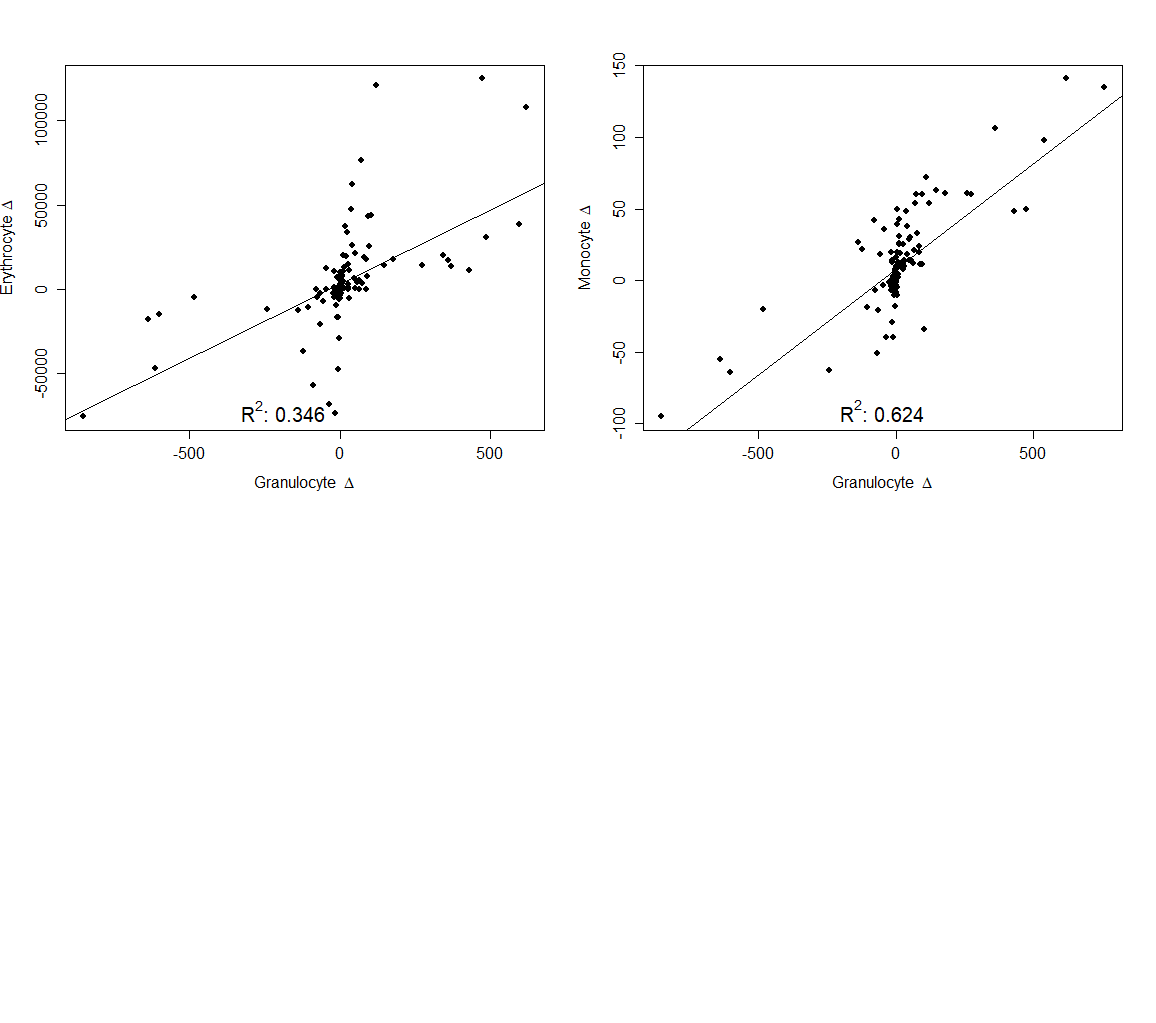

Supplement: Supplementary file 3 — Additional file 3: Correlation of paired sample differences and cell types.PNG: Scatter plots comparing the granulocyte Δ with the erythrocyte Δ and the granulocyte Δ with the monocyte Δ, respectively. Granulocytes and monocytes exhibit a stronger correlation (R2 = 0,624) vs. granulocytes and erythrocytes (R2 = 0,346) suggesting that a greater concordance is seen for cells of similar types. Δ: pair-wise difference. [file 13054_2021_3715_MOESM3_ESM.png]

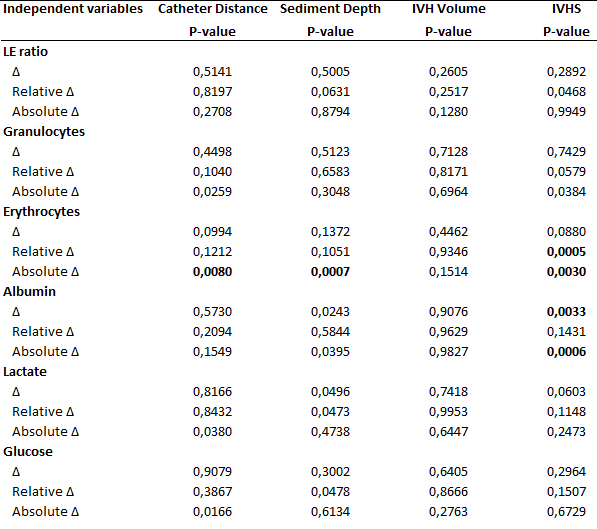

Supplement: Supplementary file 4 — Additional file 4: Computed tomography (CT)-related predictors of change between pair samples.PNG: Table with univariate mixed model linear regression analyses. The results of p < .01 are bolded. CT parameters appear most correlated with changes in erythrocytes, but not with direction of change. IVH volume, sediment depth, catheter distance, and IVHS are defined in the methods section. Δ: pair-wise difference, including absolute and relative derivatives. IVHS: intraventricular hemorrhage score. IVH: intraventricular hemorrhage. [file 13054_2021_3715_MOESM4_ESM.png]
